# Supplementary material for: Identification of distinct tumor cell populations and key genetic mechanisms through single cell sequencing in hepatoblastoma
Source: Commun Biol. 2021 Sep 8;4:1049. doi: 10.1038/s42003-021-02562-8 (PMC8426487; doi:10.1038/s42003-021-02562-8)
Supplement: Supplementary file 2 — Supplementary Information [file 42003_2021_2562_MOESM2_ESM.pdf]

Supplementary Information

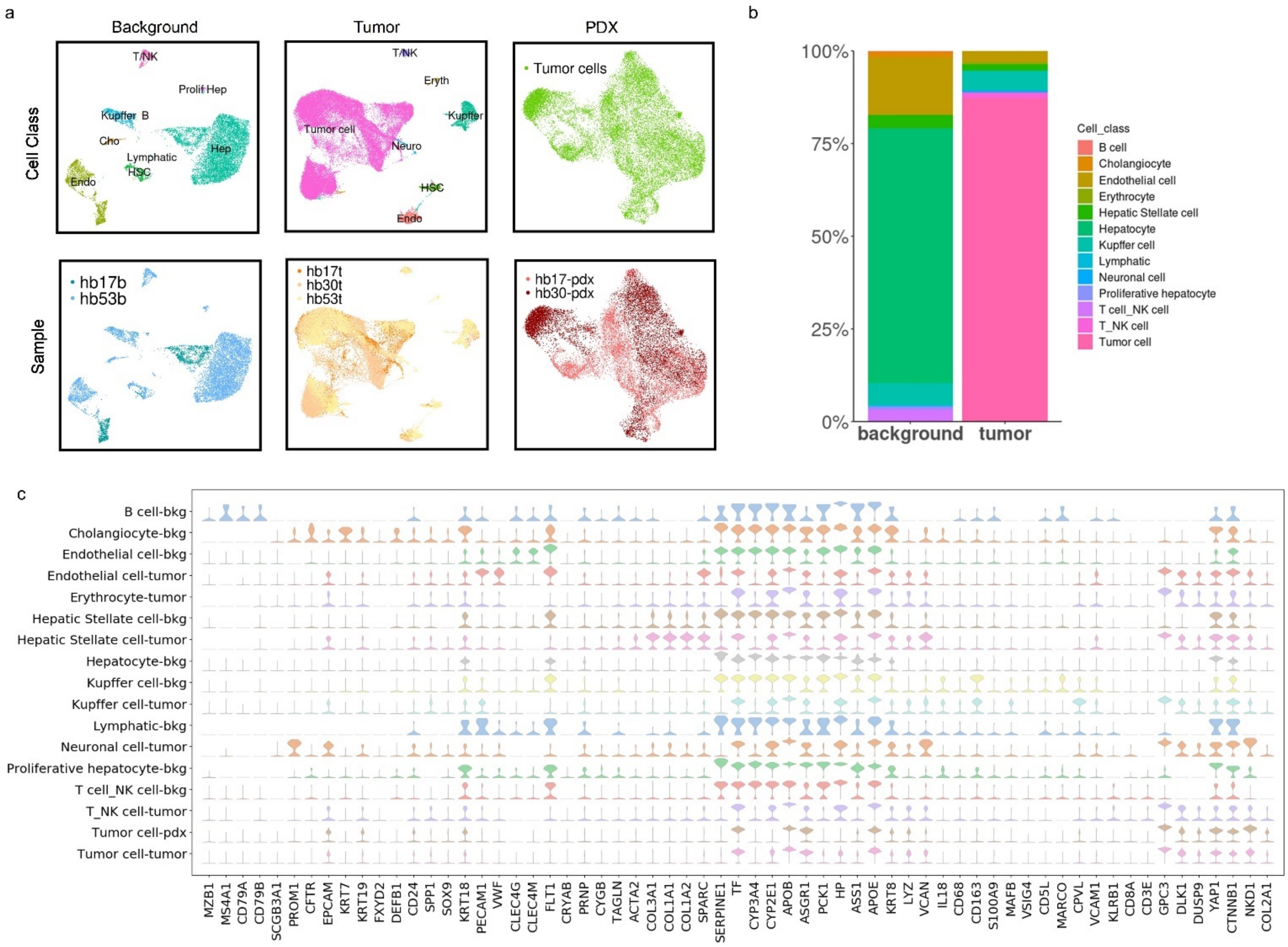

Figure S1. Defining liver and tumor cell clusters. a. The UMAP visualization showed distributions of identified cell classes (Top) and samples (Bottom) for integrated data of each sample group. b. Cell proportions in background liver and tumor were shown on the bar plot. c. The Violin plot showed expression levels (log_2(TPM+1)) of important cell markers across cell classes and samples.

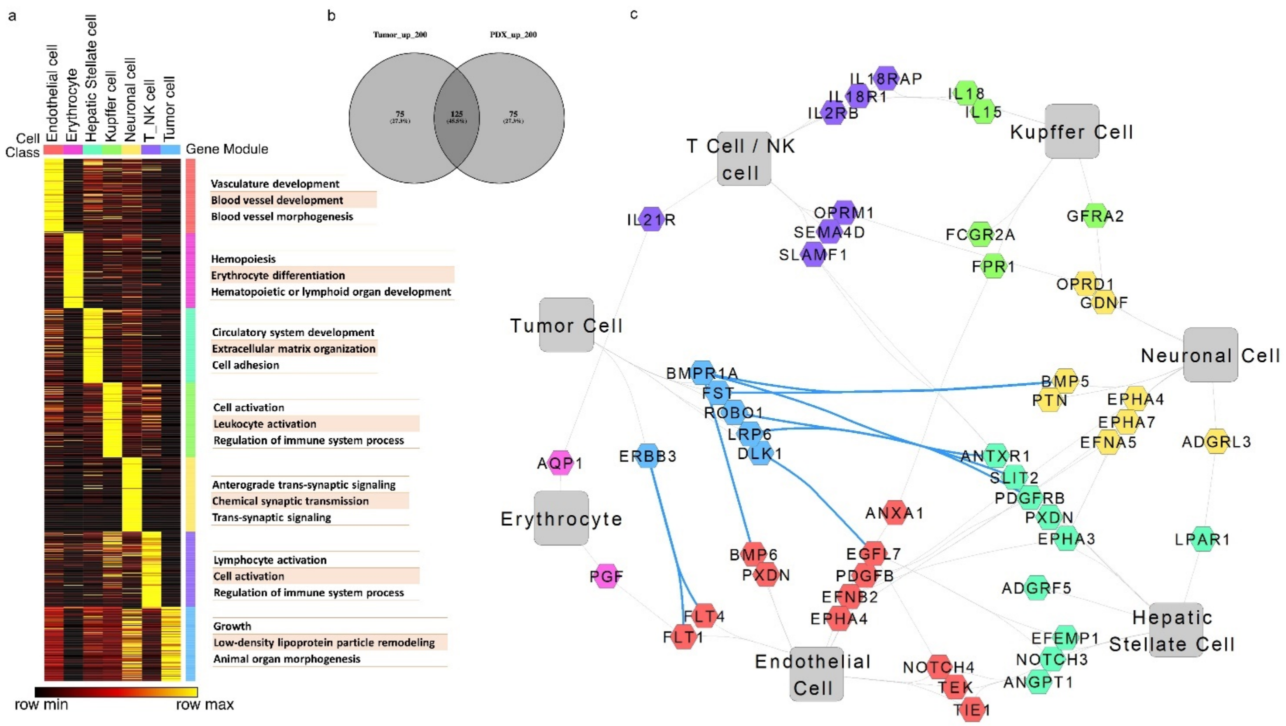

Figure S2. Tumor microenvironment molecular signatures and interactions. a. 200 most upregulated genes for each cell type in the tumor were shown in the heatmap, together with top 3 biological processes enriched in Gene Ontology (GO:Biological Process) using ToppGene. b. Venn diagram to show overlapping expression between tumor and PDX. 125 genes overlap among top 200 DEGs of comparisons against background hepatocytes in Figure 4B. c. Ligands and receptors for gene modules in (a) were selected and gene interaction network was drawn using ToppCluster. Interactions between tumor cells and other microenvironment cells were shown using thicken blue edges.

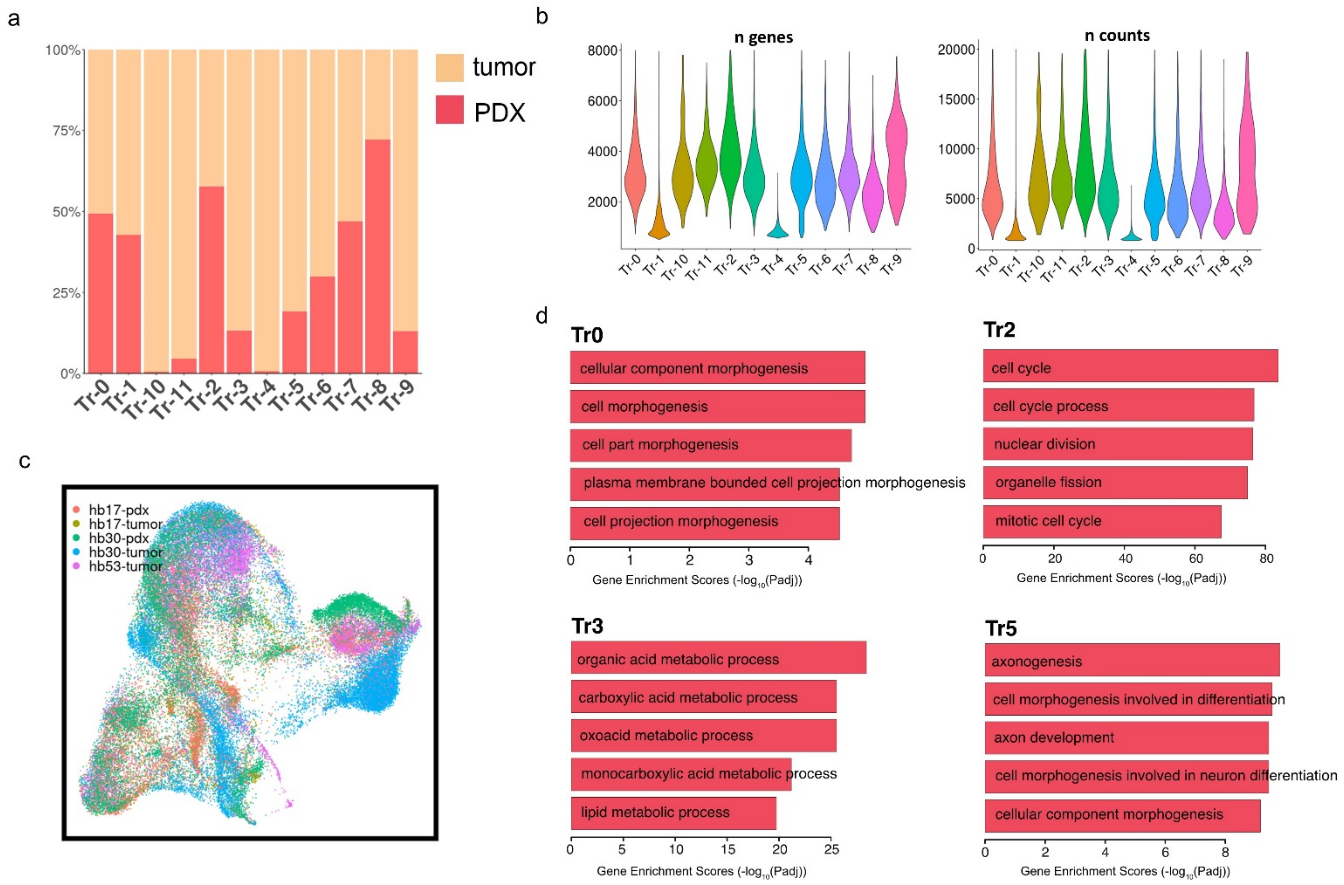

Figure S3. Tumor cluster characterization and upregulated pathways. a. Barplot depicts distributions of sample groups in each tumor cluster. b. Violin plots show number of detected genes (left) and number of detected UMIs in each tumor cluster. Tr-1 and Tr-4 are 2 clusters with low number of detected genes and UMIs. c. UMAP visualization of localization of cells from each patient tumor or PDX sample to show distribution and contribution to tumor subclusters. d. Upregulated pathways were shown for 4 critical clusters (Tr0, Tr2, Tr3 and Tr5). Top 200 differentially expressed genes were used for enrichment in ToppGene and 5 most significantly enriched pathways in gene ontology (Biological Process) were selected.

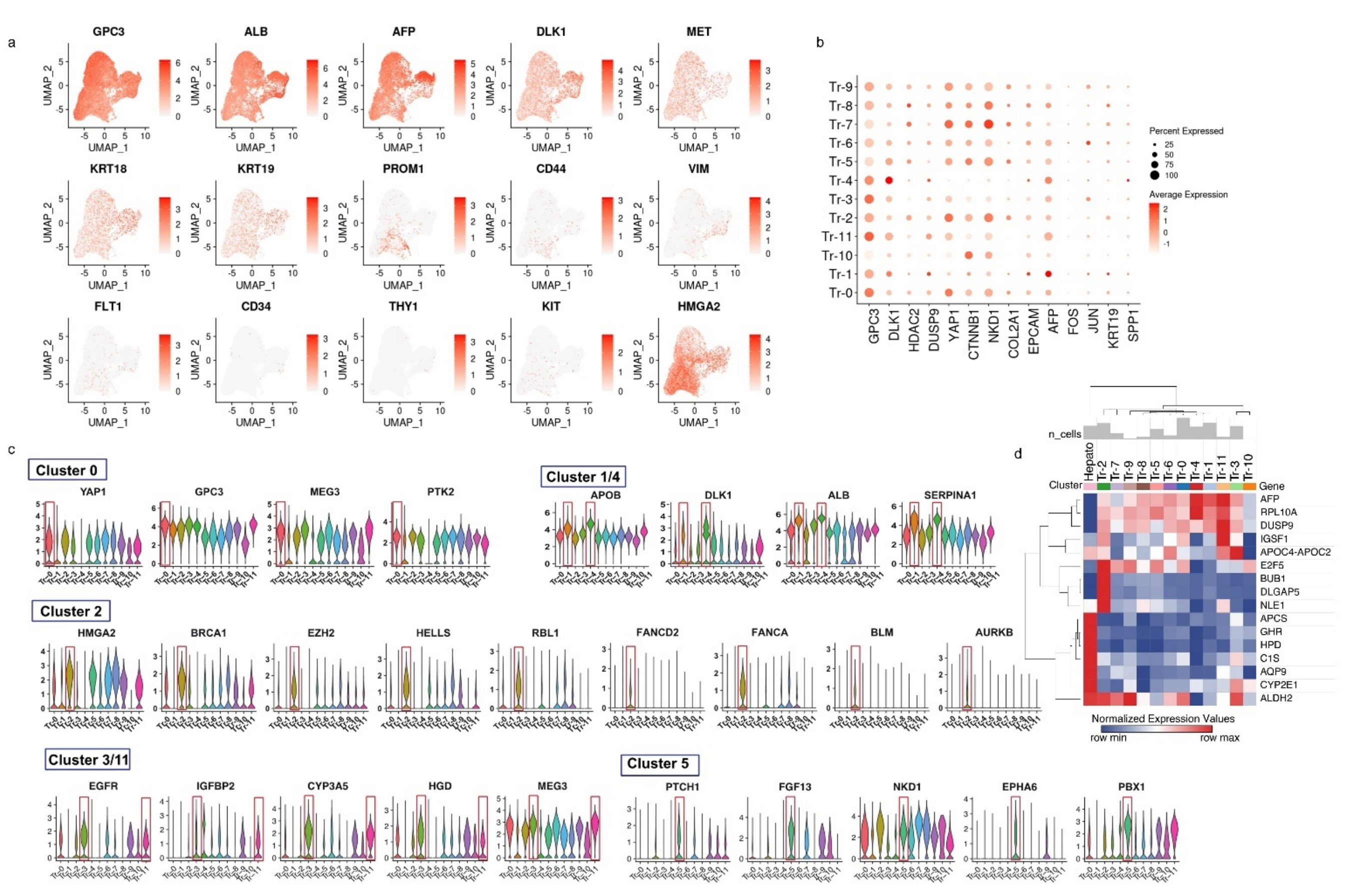

Figure S4. Differential expression in individual tumor subclusters. a. The UMAP visualization shows expression levels of 14 important genes in HB tumor development. b. Critical genes in tumor development are shown on dot plot. Mean scaled expression levels are represented by node color and proportions of expressing cells are represented by node size. c. Violin plots of differentially expressed genes elevated in key tumor subclusters (Tr0, Tr4/1, Tr2, Tr3/11, Tr5) d. Heatmap of Cairo 16 gene signature in tumor clusters compared to background hepatocytes (asterisk).

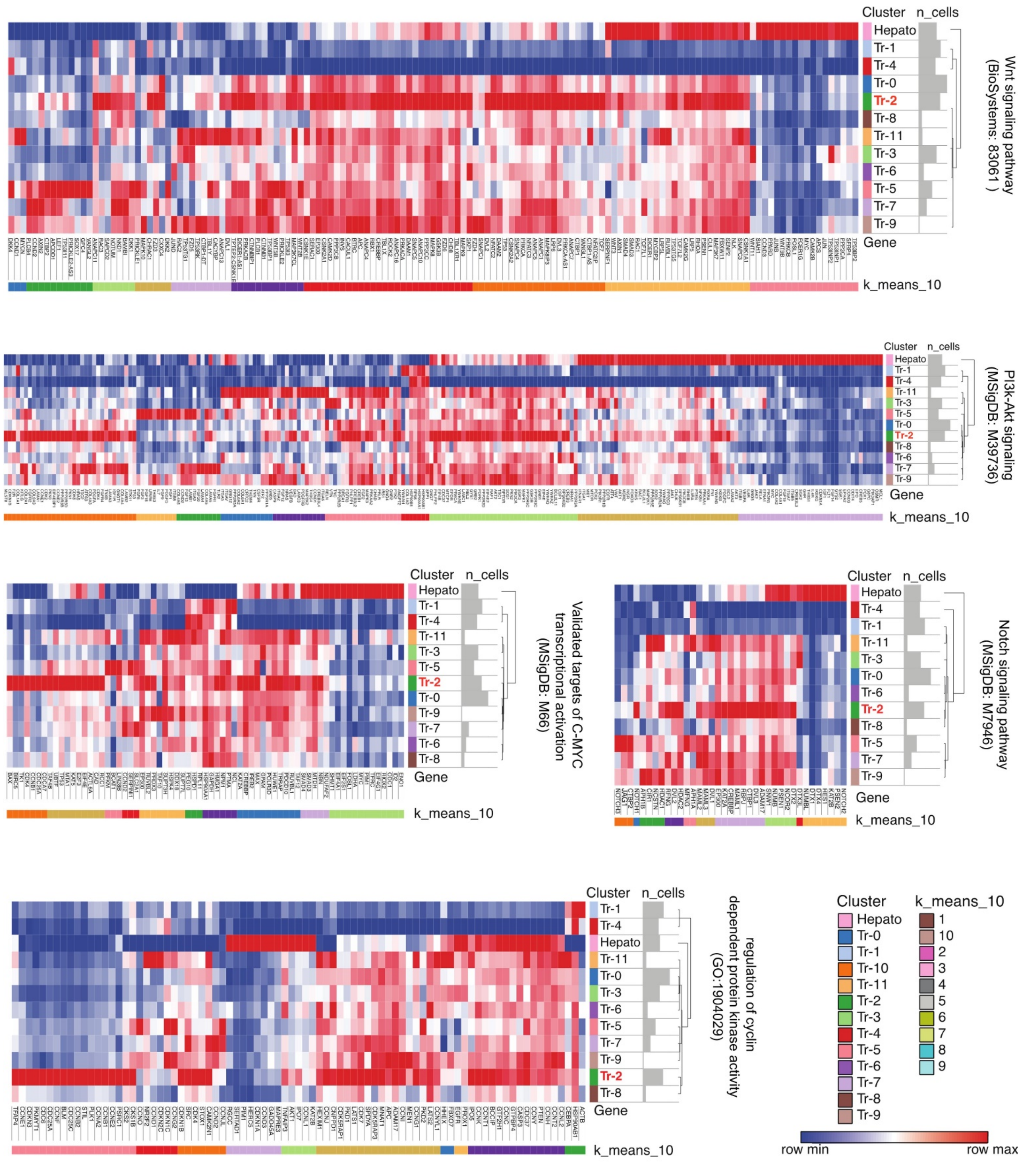

Figure S5. Tumor-associated pathways across clusters. Critical Hepatoblastoma related pathways are shown, including *WNT*, *PI3K/AKT*, *NOTCH*, and *C-MYC* signaling pathways and

cyclin dependent protein kinase activity. Hepatocytes in background liver is shown as reference. Lowly expressed genes (maximal column levels less than 0.5) were removed from the heatmap and the rest of genes were clustered using k-means clustering strategy (k=10). Hierarchical clustering was applied for rows. Tr2 is highlighted in each heatmap.

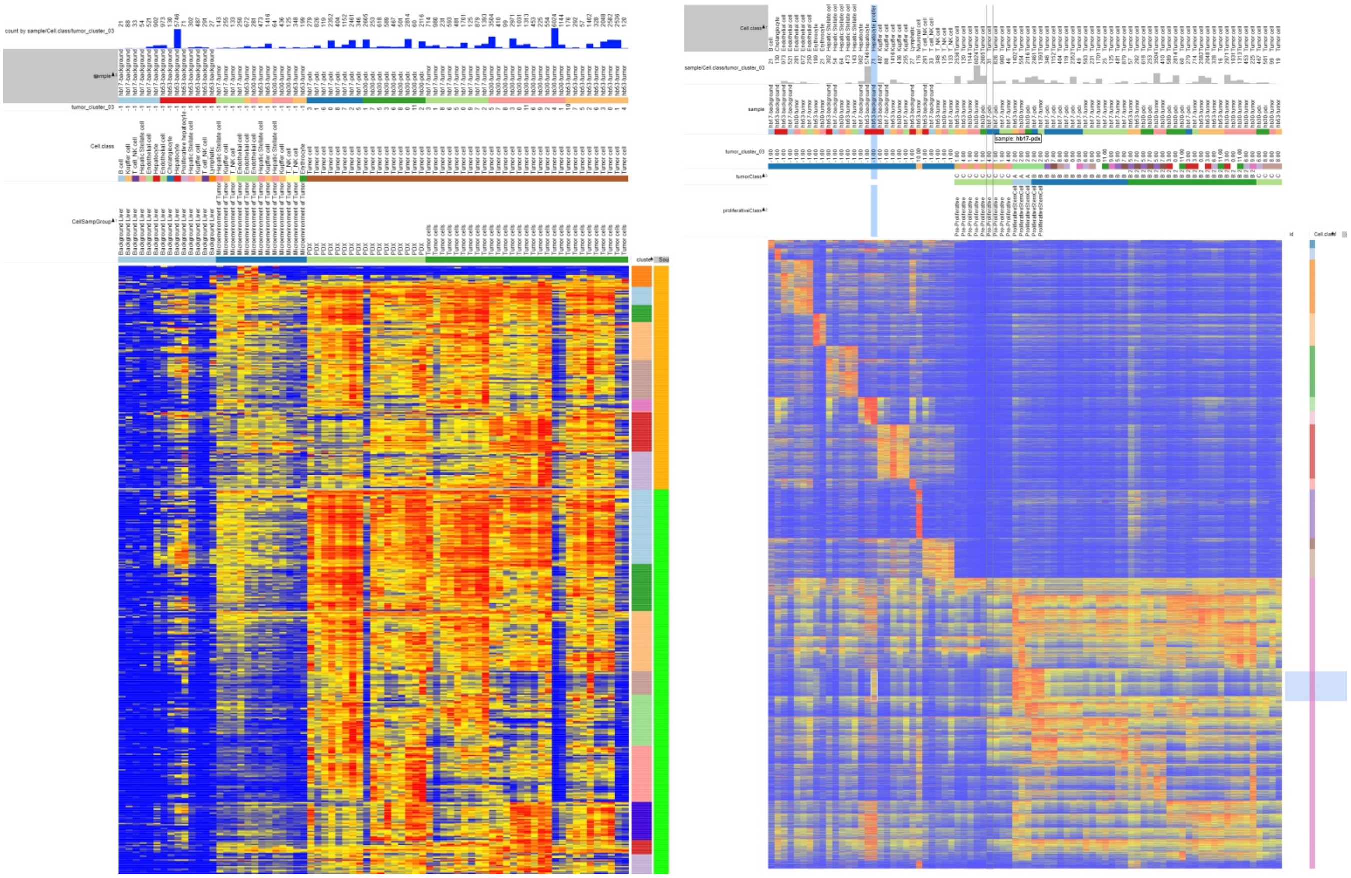

Figure S6. Individual Tumor Variations. Heatmaps of the top 200 differentially expressed genes per comparison group were generated to show similarities and differences in all tumor, background, and PDX samples used in this analysis. Left heatmap shows core tumor signature genes. Right heatmap shows expression overview organized by sample, sample group, and cell class to show tumor signatures and sample to sample variations.

Supp Table 1. Primers used for qRT-PCR.

**Primers**		
GPC3	Qiagen	PPH11457B
Axin2	Qiagen	PPH02750F
SHH	Qiagen	PPH02405A
YAP1	Qiagen	PPH13459A
Beta-Catenin	Qiagen	PPH00643F
DLK1	Qiagen	PPH00745C
IGF2	Qiagen	PPH00168B
FancD2	Qiagen	PPH14413A
GAPDH	Qiagen	PPH00150F

Supp Table 2. Antibodies used for immunostaining and western blot.

**Antibodies**			
GPC3	Roche	790-4564	PMID: 24140348
GPC3	Abcam	ab207080	PMID: 31115570
Ki67	Thermo Fisher	ma5-14520	PMID: 27446423
GAPDH	Fitzgerald	10R-G109A	PMID: 25292196
CD34	Abcam	ab8536	PMID:32102682
Cyclin D1	Roche	790-4508	PMID: 33444078
YAP	Abcam	ab52771	PMID: 32226522
EZH2	Cell Signaling	3147	PMID: 33102691
HRP-anti mouse	Biorad	1706516	PMID: 30256265
HRP-anti rabbit	Biorad	1706515	PMID: 30256265
