# Supplementary material for: Identification of distinct tumor cell populations and key genetic mechanisms through single cell sequencing in hepatoblastoma
Source: Commun Biol. 2021 Sep 8;4:1049. doi: 10.1038/s42003-021-02562-8 (PMC8426487; doi:10.1038/s42003-021-02562-8)
Supplement: Supplementary file 3 — Description of Additional Supplementary Files [file 42003_2021_2562_MOESM3_ESM.rtf]

1	Description of Additional Supplementary Files
2
3	Supplementary Data 1: RNA sequencing quality. Counts, UMI numbers, and mitochondrial genes UMIs

4	Supplementary Data 2: Marker genes.
5	·	(Sheet 1): Markers from Aizarani et al. (Aizarani, Nadim, et al. "A human liver cell atlas reveals
6	heterogeneity and epithelial progenitors." Nature 572, 199 – 204 (2019).)
7	·	(Sheet 2): S genes and G2M genes used to calculate cell cycle scores in Fig.4.

8	Supplementary Data 3: Cell annotations. Cell metadata after integration

9	Supplementary Data 4: Gene modules of cell types within each sample group (background liver, tumor
10	and PDX). Each cell type was compared to the rest of cells in that sample group or rest of cells in all
11	integrated data.
12	Student t tests were used and scores were calculated. Gene modules with top 200 most significantly
13	upregulated genes were generated. Difference of normalized expression values between the selected
14	cell type and rest of cells were shown. (See more details in
15	https://toppcell.cchmc.org/biosystems/go/index3/OncoMap).

16	Supplementary Data 5: Differential expression analysis between tumor cells in tumor and hepatocytes
17	in background liver (Student t test; Scanpy). Note: pts: percentage of cells in positive cell group with
18	non-zero expression levels; pts_rest: percentage of rest of cells with non-zero expression levels.

19	Supplementary Data 6: Single-cell RNA-seq analysis (counts and features). Tumor cluster annotations

20	Supplementary Data 7: Gene modules of tumor clusters within tumor and PDX. Each tumor cluster was
21	compared to the rest of cells in that sample group.
22	Student t tests were used and scores were calculated. Gene modules with top 200 most significantly
23	upregulated genes were generated. Difference of normalized expression values between the selected
24	cell type and rest of cells were shown. (See more details in
25	https://toppcell.cchmc.org/biosystems/go/index3/OncoMap)

26	Supplementary Data 8: qRT-PCR data for GPC3, DLK1 and IGF2.

27	Supplementary Data 9: qRT-PCR data for Axin2, Beta-catenin, FANCD2, GPC3, SHH, Wnt3a, and YAP1.

28	Supplementary Data 10: Original western blot images.
