# Supplementary figures and images for: Identification of distinct tumor cell populations and key genetic mechanisms through single cell sequencing in hepatoblastoma
Source: Commun Biol. 2021 Sep 8;4:1049. doi: 10.1038/s42003-021-02562-8 (PMC8426487; doi:10.1038/s42003-021-02562-8)

GPC3 ab207080

18B 18T 18F0 18F1 18F2 18F3 18F4 18F5 18F6

Blot 4  
7/9/19

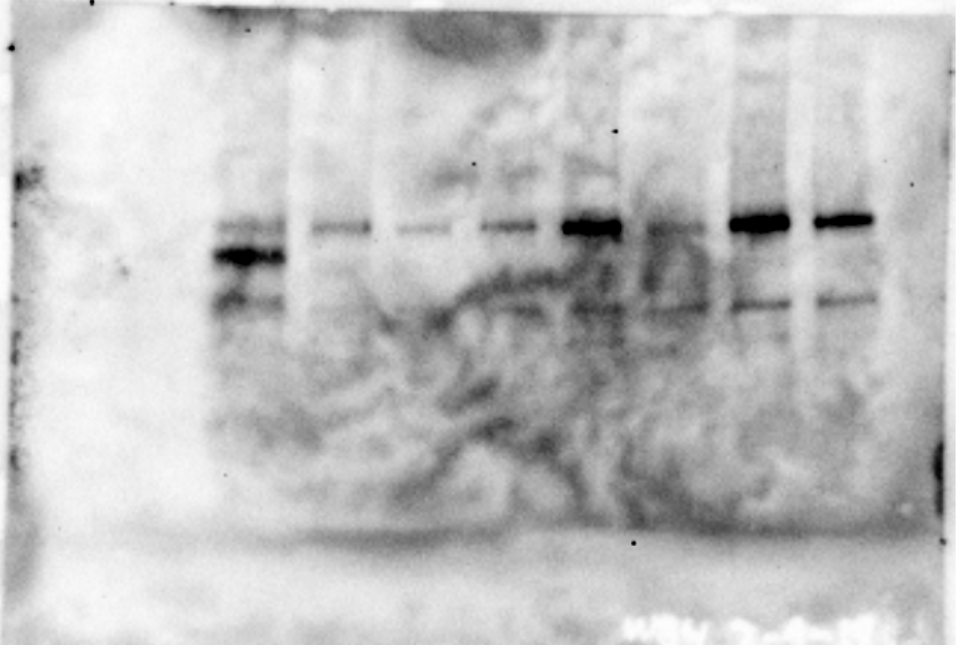

70 kDa  
50 kDa  
40 kDa

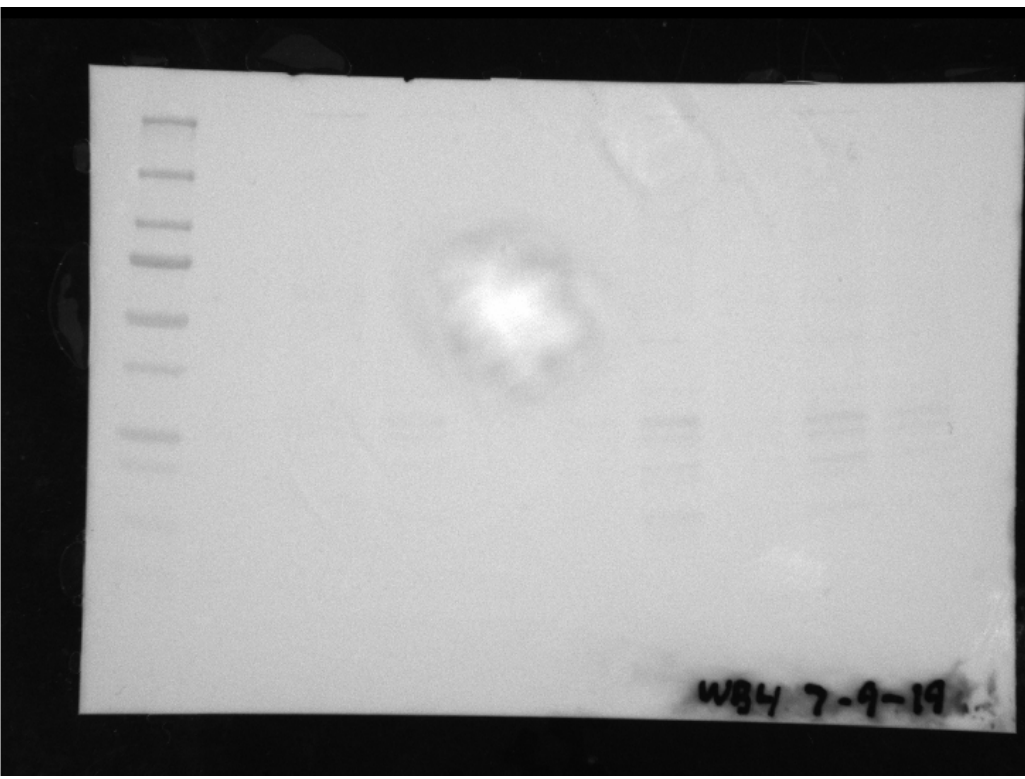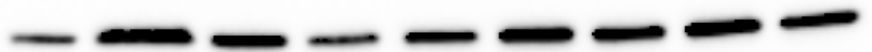

GAPDH  
10R-G109a

GPC3 ab207080

17B 17T F0 F1 F2 F3 F4 F5 F6

Blot 5  
7/22/19

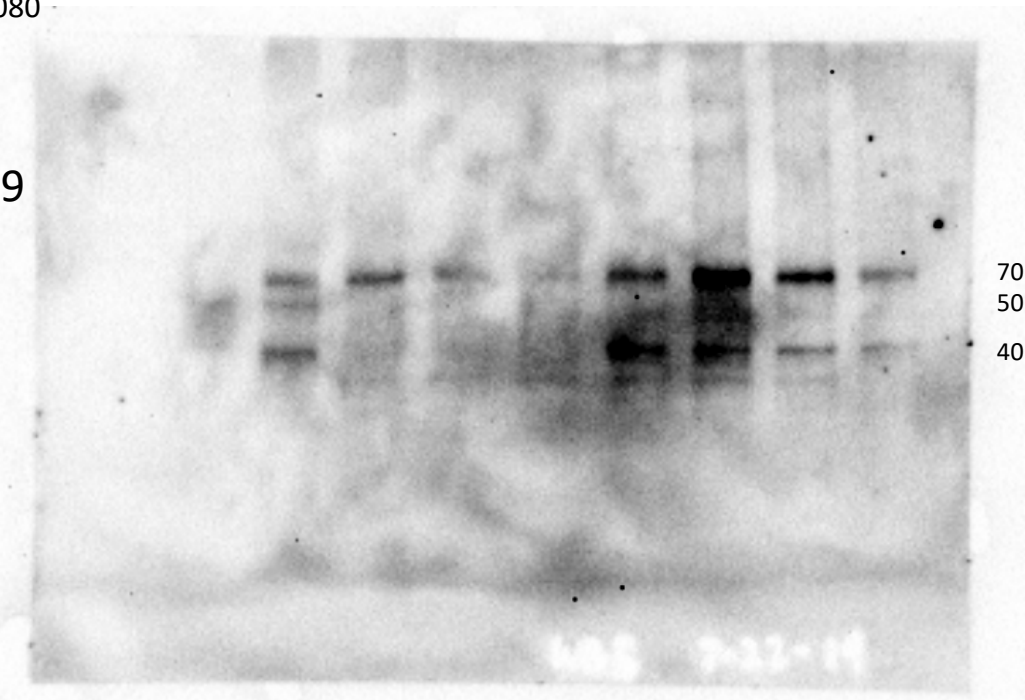

70 kDa  
50 kDa  
40 kDa

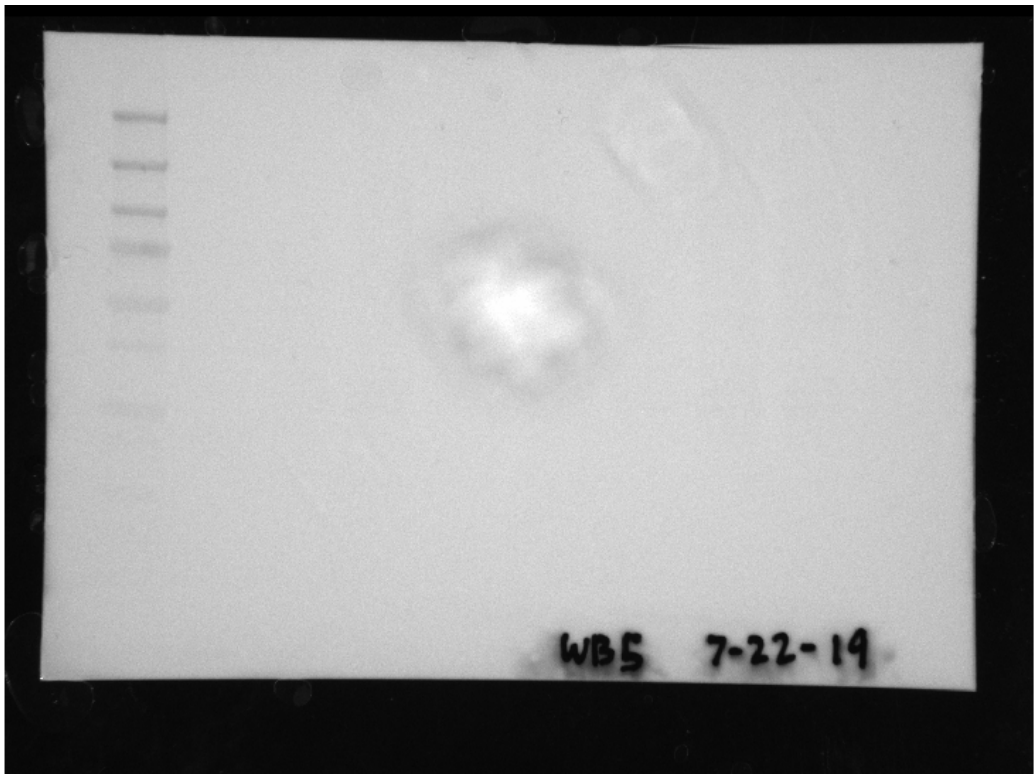

WB5 7-22-19

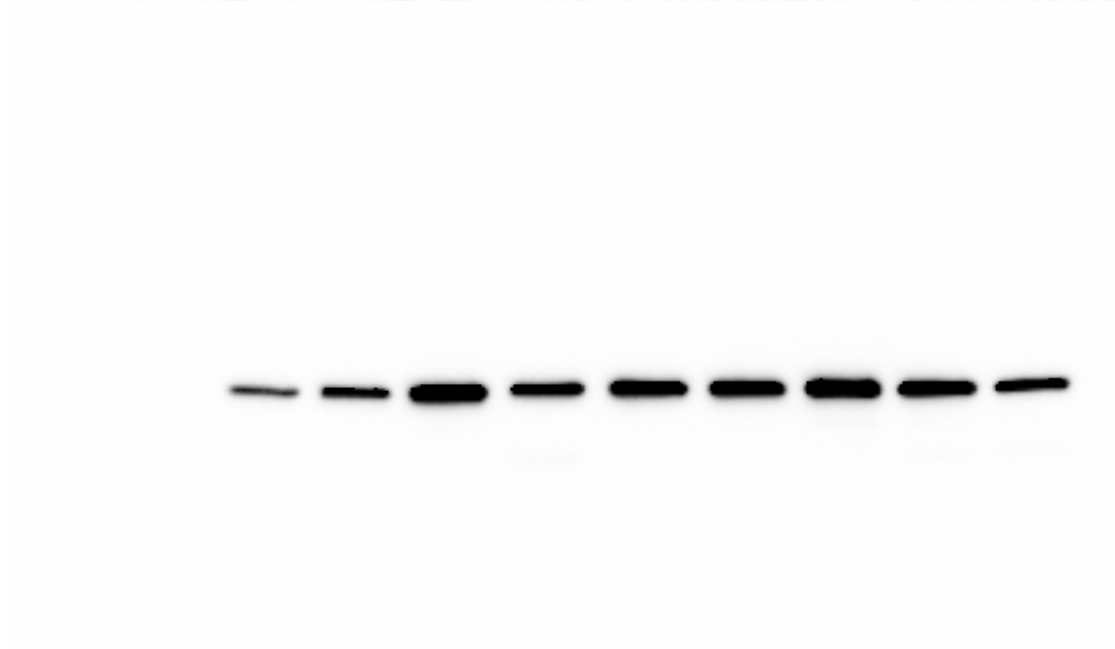

GAPDH  
10R-G109a

Supplement: Supplementary file 13 — Supplementary Data 10 [file 42003_2021_2562_MOESM13_ESM.pdf]
