# Supplementary material for: Identification of distinct tumor cell populations and key genetic mechanisms through single cell sequencing in hepatoblastoma
Source: Commun Biol. 2021 Sep 8;4:1049. doi: 10.1038/s42003-021-02562-8 (PMC8426487; doi:10.1038/s42003-021-02562-8)
Supplement: Supplementary file 14 — Reporting Summary [file 42003_2021_2562_MOESM14_ESM.pdf]

nature research

Corresponding author(s): Alexander Bondoc

Last updated by author(s): Jul 22, 2021

Reporting Summary

Nature Research wishes to improve the reproducibility of the work that we publish. This form provides structure for consistency and transparency in reporting. For further information on Nature Research policies, see our Editorial Policies and the Editorial Policy Checklist.

Statistics

For all statistical analyses, confirm that the following items are present in the figure legend, table legend, main text, or Methods section.

n/a	Confirmed	
☐	☒	The exact sample size (*n*) for each experimental group/condition, given as a discrete number and unit of measurement
☐	☒	A statement on whether measurements were taken from distinct samples or whether the same sample was measured repeatedly
☐	☒	The statistical test(s) used AND whether they are one- or two-sided<br>*Only common tests should be described solely by name; describe more complex techniques in the Methods section.*
☐	☒	A description of all covariates tested
☒	☐	A description of any assumptions or corrections, such as tests of normality and adjustment for multiple comparisons
☐	☒	A full description of the statistical parameters including central tendency (e.g. means) or other basic estimates (e.g. regression coefficient) AND variation (e.g. standard deviation) or associated estimates of uncertainty (e.g. confidence intervals)
☒	☐	For null hypothesis testing, the test statistic (e.g. *F*, *t*, *r*) with confidence intervals, effect sizes, degrees of freedom and *P* value noted<br>*Give P values as exact values whenever suitable.*
☒	☐	For Bayesian analysis, information on the choice of priors and Markov chain Monte Carlo settings
☐	☒	For hierarchical and complex designs, identification of the appropriate level for tests and full reporting of outcomes
☐	☒	Estimates of effect sizes (e.g. Cohen's *d*, Pearson's *r*), indicating how they were calculated

Our web collection on statistics for biologists contains articles on many of the points above.

Software and code

Policy information about availability of computer code

Data collection	NovaSeq 6000, Trimmimatic, HiSat2 aligner, and Kallisto for bulk RNA sequencing, 3'v3 10x gemonics, NovaSeq 6000, Cell Ranger v3.1.0 for single cell RNA sequencing
Data analysis	R package Seurat v3.1.0, Scanpy, IntegrateData in Seurat, scVelo, Toppcell,Toppcluster, Cytoscape

For manuscripts utilizing custom algorithms or software that are central to the research but not yet described in published literature, software must be made available to editors and reviewers. We strongly encourage code deposition in a community repository (e.g. GitHub). See the Nature Research guidelines for submitting code & software for further information.

Data

Policy information about availability of data

All manuscripts must include a data availability statement. This statement should provide the following information, where applicable:

- Accession codes, unique identifiers, or web links for publicly available datasets
- A list of figures that have associated raw data
- A description of any restrictions on data availability

Sequencing data that support the findings in this study have been assigned Gene Expression Omnibus accession number GSE180666 and published on Github (https://github.com/KANG-BIOINFO/scRNA-seq_Hepatoblastoma). Figures 1-7, supplemental S1-S6 contain bulk or single cell RNA sequencing data with associated GEO files.

Field-specific reporting

Please select the one below that is the best fit for your research. If you are not sure, read the appropriate sections before making your selection.

☒ Life sciences ☐ Behavioural & social sciences ☐ Ecological, evolutionary & environmental sciences

For a reference copy of the document with all sections, see nature.com/documents/nr-reporting-summary-flat.pdf

Life sciences study design

All studies must disclose on these points even when the disclosure is negative.

Sample size

Sample size was determined by availability of human hepatoblastoma tumor tissue and background liver from patients with IRB approval and patient consent as well as successful generation of patient derived xenograft models from primary tumor.

Data exclusions

No data were excluded.

Replication

Replication was confirmed by examining gene expression, protein, and histologic features of multiple human tumors, as well as RNA sequencing of multiple HB tumor, background and PDX samples. The single cell RNA sequencing was not repeated in multiple human tumors at this time, but will be verified in the future.

Randomization

Randomization was not used as samples were not separated into groups.

Blinding

Investigators were not blinded since background and tumor samples were collected from patients, analyzed, and compared without separation into groups or treatments.

Reporting for specific materials, systems and methods

We require information from authors about some types of materials, experimental systems and methods used in many studies. Here, indicate whether each material, system or method listed is relevant to your study. If you are not sure if a list item applies to your research, read the appropriate section before selecting a response.

Materials & experimental systems

n/a	Involved in the study	
☐	☒	Antibodies
☒	☐	Eukaryotic cell lines
☒	☐	Palaeontology and archaeology
☐	☒	Animals and other organisms
☐	☒	Human research participants
☒	☐	Clinical data
☒	☐	Dual use research of concern

Methods

n/a	Involved in the study	
☒	☐	ChIP-seq
☒	☐	Flow cytometry
☒	☐	MRI-based neuroimaging

Antibodies

Antibodies used

GPC3 Roche 790-4564 PMID: 24140348
GPC3 Abcam ab207080 PMID: 31115570
Ki67 Thermo Fisher ma5-14520 PMID: 27446423
GAPDH Fitzgerald 10R-G109A PMID: 25292196
CD34 Abcam ab8536 PMID:32102682
Cyclin D1 Roche 790-4508 PMID: 33444078
YAP Abcam ab52771 PMID: 32226522
EZH2 Cell Signaling 3147 PMID: 33102691
HRP-anti mouse Biorad 1706516 PMID: 30256265
HRP-anti rabbit Biorad 1706515 PMID: 30256265

Validation

Antibodies used were published in the following: PMID: 24140348, PMID: 31115570, PMID: 27446423, PMID: 25292196, PMID:32102682, PMID: 33444078, PMID: 32226522, PMID: 33102691, PMID: 30256265, PMID: 30256265. Manufacturer websites state antibody validation and additional publications.

Animals and other organisms

Policy information about studies involving animals; ARRIVE guidelines recommended for reporting animal research

Laboratory animals	Mus musculus, female NOD SCID GAMMA C-/-(NOD.Cg-PrkdcscidIl2rgtm1Wjl/SzJ) (NSG) mice, 6-8 weeks old
Wild animals	Study did not involve wild animals
Field-collected samples	Study did not involve field collected animals.
Ethics oversight	Approval was provided by Institutional Biosafety Committee (IBC #2019-0078), Institutional Animal Care and Use Committee (IACUC #2019-0077), and Institutional Review Board (IRB#2016-9497).

Note that full information on the approval of the study protocol must also be provided in the manuscript.

Human research participants

Policy information about studies involving human research participants

Population characteristics	All patients included in this study had liver tumors, surgical intervention at Cincinnati Children's Hospital and consented to our study.
Recruitment	Patients identified by the clinical team for recruitment into the study based on presence of liver tumor and diagnosis of hepatoblastoma/HCC. The Pediatric Surgery Research team contacts the family to determine interest and facilitate meeting with the principal investigator or clinical research coordinator to discuss the study and obtain consent.
Ethics oversight	Institutional Review Board (IRB#2016-9497)

Note that full information on the approval of the study protocol must also be provided in the manuscript.
